# Benefit of adjuvant immunotherapy in renal cell carcinoma: A myth or a reality?

**DOI:** 10.1371/journal.pone.0172341

**Published:** 2017-02-27

**Authors:** Satoru Taguchi, Sebastiano Buti, Hiroshi Fukuhara, Masafumi Otsuka, Melissa Bersanelli, Teppei Morikawa, Hideyo Miyazaki, Tohru Nakagawa, Tetsuya Fujimura, Haruki Kume, Yasuhiko Igawa, Yukio Homma

**Affiliations:** 1 Department of Urology, Graduate School of Medicine, The University of Tokyo, Tokyo, Japan; 2 Medical Oncology Unit, University Hospital of Parma, Parma, Italy; 3 Department of Pathology, Graduate School of Medicine, The University of Tokyo, Tokyo, Japan; Rutgers University, UNITED STATES

## Abstract

**Background:**

The benefit of adjuvant immunotherapy after nephrectomy in renal cell carcinoma (RCC) is controversial. The present study aimed to examine the possible benefit of adjuvant immunotherapy in various clinical settings.

**Methods:**

We retrospectively reviewed 436 patients with pT1-3N0-2M0 RCC who underwent radical or partial nephrectomy with curative intent at our institution between 1981 and 2009. Of them, 98 (22.5%) patients received adjuvant interferon-α (IFN-α) after surgery (adjuvant IFN-α group), while 338 (77.5%) did not (control group). The primary endpoint was cancer-specific survival (CSS). Univariate and multivariate analyses were conducted using log-rank tests and Cox proportional hazards models, respectively.

**Results:**

Fifty-two (11.9%) patients died from RCC with a median follow-up period of 96 months. Preliminary univariate analyses comparing CSS among treatment groups in each TNM setting revealed that CSS in the control group was equal or superior to that in the adjuvant IFN-α group in earlier stages, while the opposite trend was observed in more advanced stages. We evaluated the TNM cutoffs and demonstrated maximized benefit of adjuvant IFN-α in patients with pT2b-3cN0 (*P* = 0.0240). In multivariate analysis, ≥pT3 and pN1-2 were independent predictors for poor CSS in all patients. In the subgroups with ≥pT2 disease (*n* = 123), pN1-2 and no adjuvant treatment were significant poor prognostic factors.

**Conclusions:**

Adjuvant immunotherapy after nephrectomy may be beneficial in pT2b-3cN0 RCC. Careful consideration is, however, required for interpretation of this observational study because of its selection bias and adverse effects of IFN-α.

## Introduction

About a third of patients with localized renal cell carcinoma (RCC) treated by surgical resection will experience recurrence [1]. However, there is currently no established adjuvant treatment for patients after complete tumor resection [2–5]. A randomized trial conducted in the early 1980s comparing adjuvant radiotherapy after nephrectomy with observation showed no benefit of radiotherapy, with significantly increased post-radiation complications [2,3,6,7]. Based on promising data regarding the management of metastatic RCC, several randomized trials subsequently compared adjuvant interferon-α (IFN-α), high-dose interleukin-2 (IL-2) or cytokine combinations with observation alone in patients with locally advanced, completely resected RCC. However, none of these trials showed any benefit of adjuvant treatment in terms of time to relapse or improved survival [2,4,8–13]. Several phase III randomized controlled trials are currently investigating adjuvant treatment with tyrosine kinase or mammalian target of rapamycin inhibitors after nephrectomy in high-risk RCC [2,5,14]. The first of these “new generation” studies, the ASSURE trial, randomized 1943 patients with RCC to sunitinib, sorafenib, or placebo following complete resection, reported that adjuvant treatment with sorafenib or sunitinib did not improve relapse-free or overall survival (OS) compared with placebo [14,15]. Effective adjuvant treatment after nephrectomy, together with criteria for selecting suitable candidates, is therefore to be explored.

A recent phase III randomized trial comparing adjuvant immunotherapy with low-dose IL-2 plus IFN-α with observation alone after nephrectomy reported that pT3a (compared with other pT stages) could be a positive predictive factor in patients treated with adjuvant immunotherapy, maintaining its prognostic role in those not receiving adjuvant treatment [13].

In this context, the present study aimed to elucidate the optimal setting to maximize the benefit of adjuvant immunotherapy after nephrectomy in a Japanese population with RCC.

## Materials and methods

### Patients and treatments

**Fig 1:**
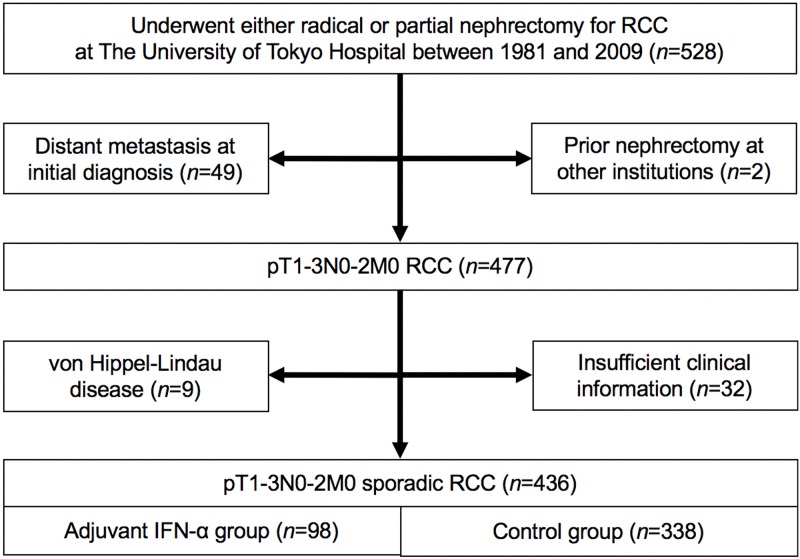
Flow chart representing the study selection process.

This retrospective study was approved by the institutional review board and was conducted in accordance with the Declaration of Helsinki. We retrospectively reviewed 528 patients with pathologically confirmed RCC who underwent radical or partial nephrectomy at The University of Tokyo Hospital between 1981 and 2009 (Fig 1). Distant metastasis at initial diagnosis (*n* = 49), prior nephrectomy at other institutions (*n* = 2), von Hippel-Lindau disease (*n* = 9) and insufficient clinical information (*n* = 32) were excluded from this analysis. A total of 436 patients with pT1-3N0-2M0 sporadic RCC who underwent either radical or partial nephrectomy with curative intent were finally reviewed, including 98 (22.5%) who received adjuvant IFN-α treatment after surgery (adjuvant IFN-α group) and 338 (77.5%) who did not (control group). Each treatment was assessed by physicians’ discretion. The adjuvant IFN-α regimens were as follows: Sumiferon^®^ (Sumitomo Dainippon Pharma, Osaka, Japan) 3–6×10^6^ IU; OIF^®^ (Otsuka Pharmaceutical, Tokyo, Japan) 5×10^6^ IU; or Intron^®^ A (Merck Sharp & Dohme, Tokyo, Japan) 3–6×10^6^ IU, injected subcutaneously two to three times per week. Pathological stage was re-evaluated according to the 7^th^ TNM classification of the Union for International Cancer Control (UICC) and the American Joint Committee on Cancer (AJCC) Guidelines [16]. This TNM re-evaluation was conducted in a comprehensive manner, based on pathology reports, medical charts, radiogram interpretation reports, and so on. Histological subtype and tumor grade were assessed according to 3^rd^ World Health Organization Classification of Tumours [17] and the Heidelberg classification [18], respectively. These are the main criteria currently used in Japan for the pathological diagnosis of RCC [19]. All patients underwent preoperative and postoperative (every 1–6 months) evaluations, including routine blood tests, chest x-rays, and computed tomography. Bone scintigraphy was performed when indicated. Postoperative monitoring included routine chest x-rays every 3 months and/or chest and abdominal computed tomography every 6 months in the first 3 years, and yearly thereafter.

### Statistical analyses

The primary endpoint was cancer-specific survival (CSS). Secondary endpoints were OS and recurrence-free survival (RFS). Survival curves were drawn using the Kaplan—Meier method. Univariate and multivariate analyses were performed using log-rank tests and Cox proportional hazards model, respectively. All statistical analyses were performed using JMP Pro version 11.0.0 (SAS Institute, Cary, NC, USA). A value of *P*<0.05 was considered significant. Follow-up information was obtained up to December 2015.

## Results

### Patient characteristics

Fifty-two (11.9%) patients died from RCC with a median follow-up period of 96 months. The patient characteristics are summarized in Table 1. pT stage, pN stage, and grade were significantly higher in the adjuvant IFN-α group, but there were no significant differences in age, sex, histological subtype, and follow-up period between the two groups. The surgical procedures were open radical nephrectomy in 253 (58.0%), open partial nephrectomy in 120 (27.5%), laparoscopic radical nephrectomy in 61 (14.0%), and laparoscopic partial nephrectomy in two (0.5%) patients; there was no difference of the surgery type among the two groups. The data on adjuvant IFN-α duration were available in 51 of 98 (52%) patients: their median IFN-α duration was 10 months (range: 1–175 months).

**Table 1:** Patient characteristics.

Parameter	Total (*n* = 436)	Adjuvant IFN-α (*n* = 98)	Control (*n* = 338)	*P*
Age at surgery, years, median (IQR)	59 (50–67)	57 (48–66)	59 (51–68)	0.0631^a^
Gender, no. (%):				0.3339^b^
Male	331 (75.9)	78 (79.6)	253 (74.9)	
Female	105 (24.1)	20 (20.4)	85 (25.1)	
pT stage, no. (%):				<0.0001*^b^
T1a	202 (47.9)	12 (12.2)	190 (56.2)	
T1b	111 (25.5)	28 (28.6)	83 (24.6)	
T2a	45 (10.3)	16 (16.3)	29 (8.6)	
T2b	9 (2.1)	4 (4.1)	5 (1.5)	
T3a	59 (13.5)	34 (34.7)	25 (7.4)	
T3b	4 (0.9)	3 (3.1)	1 (0.3)	
T3c	6 (1.4)	1 (1.0)	5 (1.5)	
pN stage, no. (%)				0.0001*^b^
N0/x	422 (96.8)	89 (90.8)	333 (98.5)	
N1-2	14 (3.2)	9 (9.2)	5 (1.5)	
Histological subtype, no. (%):				0.9325^b^
Clear cell	395 (90.6)	89 (90.8)	306 (90.5)	
Non-clear cell	41 (9.4)	9 (9.2)	32 (9.5)	
Grade				0.0212*^b^
G1	96 (22.0)	13 (13.3)	83 (24.6)	
G2	295 (67.7)	70 (71.4)	225 (66.6)	
G3	45 (10.3)	15 (15.3)	30 (8.9)	
Median follow-up, months (IQR)	96 (45–140)	96 (43–163)	96 (46–135)	0.1235^a^

IQR = interquartile range

* Statistically significant;

^a^ Student’s *t*-test;

^b^ Pearson’s χ^2^ test

### Treatment outcomes

**Fig 2:**
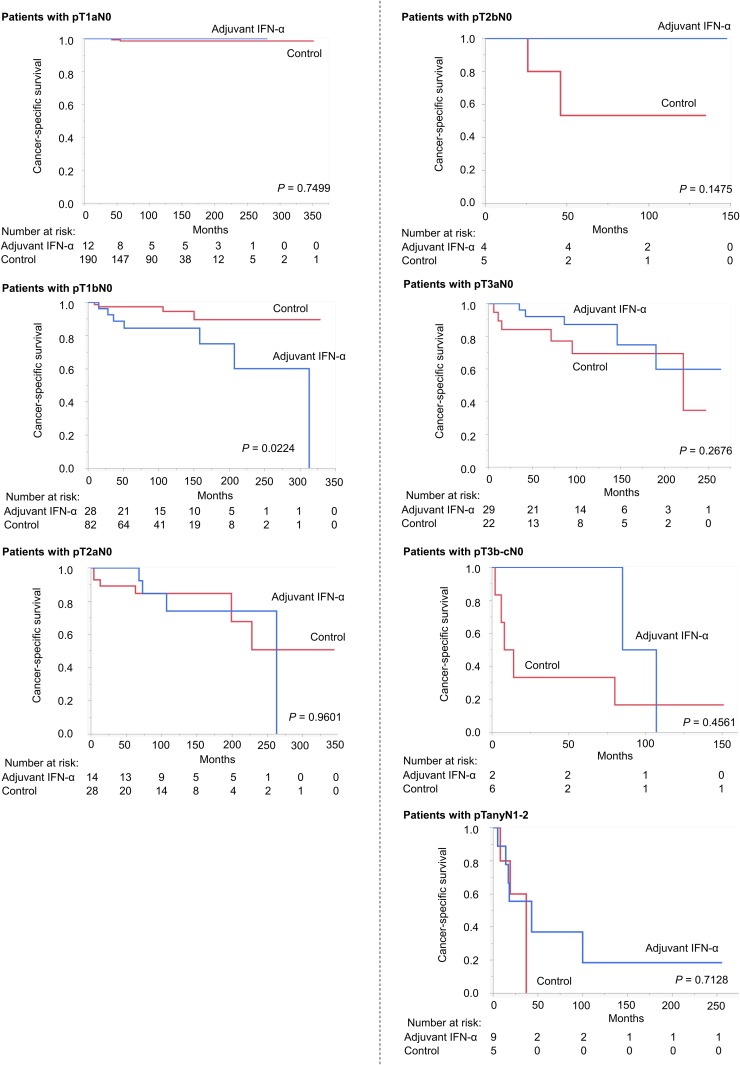
Kaplan—Meier curves depicting CSS in adjuvant IFN-α and control groups in each TNM setting. CSS in the control group was equal or superior to that in the adjuvant IFN-α group in earlier stages (pT1aN0, pT1bN0, pT2aN0), but the opposite trend was observed in more advanced stages (pT2bN0, pT3aN0, pT3b-cN0, pTanyN1-2).

**Fig 3:**
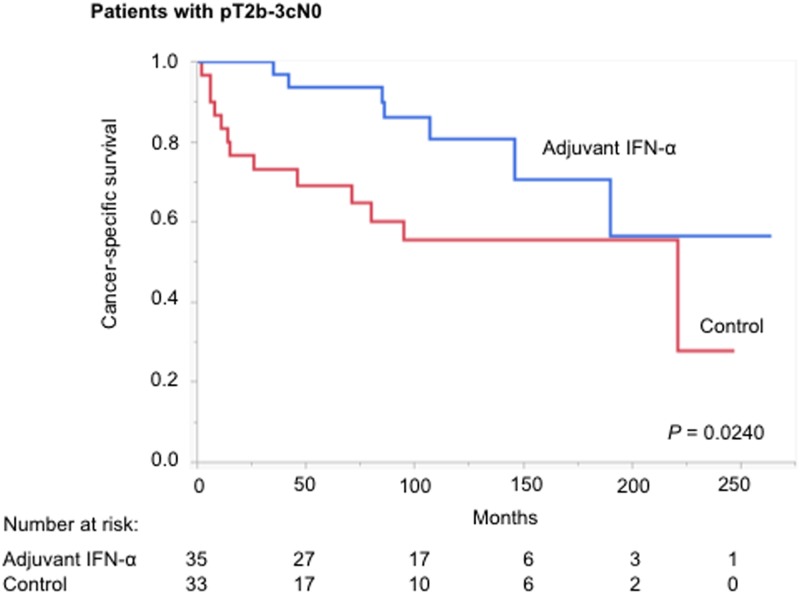
Kaplan—Meier curves depicting CSS in adjuvant IFN-α and control groups in patients with pT2b-3cN0 (*P* = 0.0240, log-rank test).

We conducted preliminary univariate analyses to compare CSS between the adjuvant IFN-α and control groups in each TNM setting (Fig 2). CSS in the control group was equal or superior to that in the adjuvant IFN-α group in earlier stages (pT1aN0, pT1bN0, pT2aN0), but the opposite trend was observed in more advanced stages (pT2bN0, pT3aNo, pT3b-cN0, pTanyN1-2). Based on these findings, we evaluated the TNM cutoffs and demonstrated that adjuvant IFN-α had maximal benefit in patients with pT2b-3cN0 (*P* = 0.0240) (Fig 3). S1 Fig presents this result in another way: CSS in patients with pT2b-3cN0 was similar to that for pT1a-2aN0 in the adjuvant IFN-α group, but significantly worse in the control group. Similar trends were observed for the secondary endpoints (OS and RFS) (S2 Fig).

In addition to TNM, histological subtype (clear cell vs. non-clear cell) and grade (G1-2 vs. G3) were also associated with CSS in univariate analysis. However, multivariate analysis only identified ≥pT3 and pN1-2 as independent predictors of poor CSS in the overall population (Table 2). For reference, multivariate analysis in the subgroup of patients with ≥pT2 disease (*n* = 123) detected pN1-2 and omission of adjuvant treatment as independent poor prognostic factors (Table 3).

**Table 2:** Univariate and multivariate analyses of cancer-specific survival in all patients (*n* = 436).

Parameter	Cutoff	Univariate	Multivariate	
		*P*	HR (95% CI)	*P*
Age at surgery	<59 years†	0.8166	Reference	0.3700
	≥59 years†		0.764 (0.417 to 1.375)	
Gender	Male	0.2279	Reference	0.5962
	Female		0.822 (0.369 to 1.646)	
pT stage	≤T2	<0.0001*	Reference	<0.0001*
	≥T3		4.338 (2.187 to 8.442)	
pN stage	N0/x	<0.0001*	Reference	0.0296*
	N1-2		3.275 (1.132 to 8.625)	
Histological subtype	Clear cell	0.0324*	Reference	0.7989
	Non-clear cell		1.121 (0.439 to 2.553)	
Grade	G1-2	<0.0001*	Reference	0.0550
	G3		2.221 (0.982 to 4.605)	
Treatment group	Adjuvant IFN-α	0.0005*	Reference	0.9073
	Control		1.040 (0.532 to 1.997)	

HR = hazard ratio; CI = confidence interval

^†^ Median;

* statistically significant

**Table 3:** Univariate and multivariate analyses of cancer-specific survival in patients with ≥pT2 disease (*n* = 123).

Parameter	Cutoff	Univariate	Multivariate	
		*P*	HR (95% CI)	*P*
Age at surgery	<59 years†	0.6429	Reference	0.2517
	≥59 years†		0.672 (0.332 to 1.323)	
Gender	Male	0.4567	Reference	0.7167
	Female		0.861 (0.355 to 1.871)	
pT stage	≤T2	0.0553	Reference	0.1182
	≥T3		1.746 (0.869 to 3.636)	
pN stage	N0/x	<0.0001*	Reference	0.0013*
	N1-2		5.163 (1.973 to 12.48)	
Histological subtype	Clear cell	0.5811	Reference	0.9870
	Non-clear cell		1.008 (0.369 to 2.430)	
Grade	G1-2	0.0727	Reference	0.3716
	G3		1.492 (0.598 to 3.328)	
Treatment group	Adjuvant IFN-α	0.2354	Reference	0.0210*
	Control		2.227 (1.128 to 4.532)	

HR = hazard ratio; CI = confidence interval

^†^ Median;

* statistically significant

## Discussion

The present study demonstrated that the benefit of adjuvant IFN-α after nephrectomy was detected in patients with pT2b-3cN0 RCC. CSS was significantly prolonged in this subgroup following adjuvant IFN-α treatment, compared with the control group. Adjuvant immunotherapy improved the prognosis of patients with pT2b-3cN0 tumors to a similar risk level to those with lower pathological stages. The CSS curves for patients with lower or higher pT were not significantly affected by adjuvant therapy, apart from a slight tendency towards a detrimental effect for pT1a-2aN0. Similar trends were also observed for the other endpoints of OS and RFS. Furthermore, although adjuvant IFN-α was not prognostic in the study population as a whole, it was a good independent prognostic factor in patients with ≥pT2 disease.

To the best of our knowledge, six previously published randomized trials have compared cytokine-based (IFN-α and/or IL-2) adjuvant treatment with observation after nephrectomy, all of which failed to show any survival benefit of adjuvant immunotherapy [8–13]. The results of previous retrospective studies assessing the efficacy of adjuvant immunotherapy were also generally disappointing [20–23]. However, unplanned subgroup analysis of a randomized trial by Passalacqua et al. reported that pT3a (compared with other pT stages) could be a positive predictive factor in patients treated with adjuvant therapy, maintaining its prognostic role in the control group [13]. The authors developed a scoring model comprising pN (N0 vs. N1-2), tumor grade (Fuhrman G1-2 vs. G3-4), pT stage (pT3a vs. others [pT1-2 & pT3b-3c] according to the 6^th^ edition of the UICC-AJCC TNM staging system [24]), and age (≤60 vs. >60 years), and observed better RFS and OS outcomes in the adjuvant-treatment arm in patients with higher scores (i.e., ≥2 vs. 0–1 factors among pN0, G1-2, pT3a, and age ≤60 years) [13]. The results of the current study were generally in accordance with this Passalacqua’s report, indicating maximal benefit of adjuvant immunotherapy in patients with pT2b-3c (around pT3a) but without nodal metastasis (pN0). It is necessary to point out that the TNM classification for pT3a has been modified over time, from being defined as the extension to “perinephric tissue, renal sinus, or contiguous into adrenal gland” (T3b for renal vein involvement) in 2002 [24], to include “perinephric tissue, renal sinus, or renal vein” (the adrenal gland involvement was attributed to T4) in 2010 [16]. In the present study, the pathological stage was revised according to the 7^th^ TNM classification.

IFN-α has established roles in the treatment of RCC in the metastatic setting [2] and malignant melanoma in both adjuvant and metastatic settings [25], but its mechanism of action has not been fully elucidated. Researchers have speculated that IFN-α may exert its antitumor efficacy mainly by indirect immunomodulatory effects, involving several mechanisms. These include an increase in tumor-infiltrating cells, decrease in circulating regulatory T cells, manifestations of autoimmunity and development of autoantibodies, changes in cytokine concentrations, modulation of signal transducer and activator of transcription (STAT) 1/STAT3 balance in tumor cells and host lymphocytes, and normalization of T cell STAT1 signaling defects in peripheral blood lymphocytes [26]. These indirect immunomodulatory effects are assumed to be enhanced under certain levels of tumor burden. Our results suggested that tumor-associated antigens might be highly presented to the systemic circulation in patients with ≥pT2b disease, which could strengthen the antitumor actions of adjuvant IFN-α (e.g., preventing further growth of micrometastases). Conversely, these indirect effects are less powerful and might thus be unable to improve the anti-tumor response in more advanced settings (pN1-2), resulting in an optimal response in patients with moderately advanced tumors (pT2b-3cN0).

According to our analysis, adjuvant IFN-α was associated with poorer CSS compared with the control group in patients with earlier stage RCC, such as pT1bN0. It might be caused by the selection bias that patients with higher grade tumors were more assigned to adjuvant IFN-α treatment. However, the previous randomized trial also reported a similar trend of poorer outcomes associated with adjuvant treatment in earlier stages, suggesting that adjuvant immunotherapy may indeed have a detrimental effect in earlier stage RCC [13]. Another randomized trial conducted in Japan also reported a similar trend, with higher RFS in the observation group compared with the interferon group in T1 or T2 subjects, but higher RFS in the interferon group over 3 years in T3 subjects, though the difference was not significant [12]. No other study of the said six trials assessing cytokine-based adjuvant therapy after nephrectomy compared treatment outcomes in a TNM subgroup [8–11]. Nevertheless, its minimal or negative impact on survival, together with its well-known adverse effects such as fatigue, headache, muscle pain, and depression, means that adjuvant IFN-α should be cautiously indicated. Attention should also be paid to the fact that these adverse effects of IFN-α are quite different from those of tyrosine kinase inhibitors including hypertension, hand-foot syndrome, rash and fatigue [15].

This study had some limitations, including its retrospective design and potential selection bias. Given the mechanism of action of immunotherapy, the results of this study might be useful for trials of emerging immune checkpoint inhibitors [5,13].

## Conclusions

The benefit of adjuvant immunotherapy was most significant in RCC patients with pT2b-3cN0. Careful consideration is, however, required for interpretation of this observational study because of its selection bias and adverse effects of IFN-α. Further studies are required to validate these results and aid the establishment of optimal patient-selection criteria for trials of adjuvant immunotherapy after nephrectomy.

## Supporting information

S1 Dataset The clinical and pathological data of all the subjects. (XLSX)

S1 Fig Kaplan—Meier curves depicting CSS in pT1a-2aN0, pT2b-3cN0, and pTanyN1-2 patients among (A) all patients, (B) adjuvant IFN-α group, and (C) control group, respectively (all *P*<0.0001, log-rank test). CSS in pT2b-3cN0 was similar to that in pT1a-2aN0 in the adjuvant IFN-α group, but significantly worse in the control group.
(TIF)

S2 Fig Kaplan—Meier curves depicting OS in pT1a-2aN0, pT2b-3cN0, and pTanyN1-2 patients among (A) all patients, (B) adjuvant IFN-α group, and (C) control group, respectively (ditto with RFS). Similar trends to CSS were observed for both OS and RFS.
(TIF)
